# A family history of breast cancer will not predict female early onset breast cancer in a population-based setting

**DOI:** 10.1186/1471-2407-8-203

**Published:** 2008-07-23

**Authors:** Geertruida H de Bock, Catharina E Jacobi, Caroline Seynaeve, Elly MM Krol-Warmerdam, Jannet Blom, Christi J van Asperen, Cees J Cornelisse, Jan GM Klijn, Peter Devilee, Rob AEM Tollenaar, Cecile TM Brekelmans, Johannes C van Houwelingen

**Affiliations:** 1 Department of Epidemiology, Groningen University Medical Center, Groningen, The Netherlands; 2 Department of Medical Decision Making, Leiden University Medical Center, Leiden, The Netherlands; 3 Department of Medical Oncology, Erasmus Medical Center-Dr Daniel den Hoed Cancer Center, Rotterdam, The Netherlands; 4 Department of Surgery, Leiden University Medical Center, Leiden, The Netherlands; 5 Department of Clinical Genetics, Leiden University Medical Center, Leiden, The Netherlands; 6 Department of Pathology, Leiden University Medical Center, Leiden, The Netherlands; 7 Department of Human Genetics, Leiden University Medical Center, Leiden, The Netherlands; 8 Department of Medical Statistics, Leiden University Medical Center, Leiden, The Netherlands

## Abstract

**Background:**

An increased risk of breast cancer for relatives of breast cancer patients has been demonstrated in many studies, and having a relative diagnosed with breast cancer at an early age is an indication for breast cancer screening. This indication has been derived from estimates based on data from cancer-prone families or from BRCA1/2 mutation families, and might be biased because BRCA1/2 mutations explain only a small proportion of the familial clustering of breast cancer. The aim of the current study was to determine the predictive value of a family history of cancer with regard to early onset of female breast cancer in a population based setting.

**Methods:**

An unselected sample of 1,987 women with and without breast cancer was studied with regard to the age of diagnosis of breast cancer.

**Results:**

The risk of early-onset breast cancer was increased when there were: (1) at least 2 cases of female breast cancer in first-degree relatives (yes/no; HR at age 30: 3.09; 95% CI: 128-7.44), (2) at least 2 cases of female breast cancer in first or second-degree relatives under the age of 50 (yes/no; HR at age 30: 3.36; 95% CI: 1.12–10.08), (3) at least 1 case of female breast cancer under the age of 40 in a first- or second-degree relative (yes/no; HR at age 30: 2.06; 95% CI: 0.83–5.12) and (4) any case of bilateral breast cancer (yes/no; HR at age 30: 3.47; 95%: 1.33–9.05). The positive predictive value of having 2 or more of these characteristics was 13% for breast cancer before the age of 70, 11% for breast cancer before the age of 50, and 1% for breast cancer before the age of 30.

**Conclusion:**

Applying family history related criteria in an unselected population could result in the screening of many women who will not develop breast cancer at an early age.

## Background

Breast cancer is the most commonly diagnosed cancer in women and the second leading cause of cancer death [1]. An increased risk of breast cancer for relatives of breast cancer patients has been demonstrated in many studies [2,3]. As physicians and the general population are becoming more aware of this increased risk, the demand for referring healthy women with a family history of breast cancer for intensive screening or genetic testing, is rising.

In most guidelines the prerequisite for starting breast cancer surveillance before the age of 50 is at least one first-degree relative diagnosed with breast cancer under the age of 40 [4,5] or 50 [6,7]. Additional criteria are: more than one relative with breast cancer, relatives with bilateral breast cancer, ovarian cancer, male breast cancer, or prostate cancer before the age of 60 [8]. These criteria have been derived from estimates based on data from cancer-prone families or from BRCA1/2 mutation families. As BRCA1/2 mutations explain only a small proportion of the familial clustering of breast cancer [9], estimates based on these high-risk families may thus have limited value for the prediction of the age of onset of breast cancer in the general population. Therefore, empirical data of risks for family members of unselected cases of breast cancer are needed to improve current guidelines.

In this study, we used data regarding sisters of recently diagnosed breast cancer patients. These patients were unselected for their age at breast cancer diagnosis, and their family history. The aim of this study was to determine the predictive value of a family history of cancer with regard to early onset of female breast cancer in a population-based setting.

## Methods

### Design

We studied the predictive value of a family history of cancer with regard to early onset of breast cancer in a large group of women with and without breast cancer. These women were unselected for having breast cancer, but they all had a family history of breast cancer, having at least one sister with breast cancer, as they were selected via their affected sister. These affected sisters (the index patients) had recently been diagnosed with breast cancer and originated from a cohort of breast cancer patients who were unselected for age of breast cancer or family history.

### Index patients

Index patients (affected sisters) had a recent diagnosis of primary invasive breast cancer or DCIS (ductal carcinoma in situ) and were consecutively asked to participate in a population-based study "PROSPECT" from 1996 to 2002. This population-based study was carried out at the Leiden University Medical Center, Department of Surgery, at the Diaconessenhuis, Leiden, and at the Erasmus Medical Center-Daniel den Hoed Cancer Center in Rotterdam, Departments of Medical Oncology, Radiotherapy and Surgery. At the Leiden University Medical Center and the Diaconessenhuis, all patients recently diagnosed with breast cancer (n = 514) were asked to participate in this study, irrespective of age. The participation rate was 92% (n = 471). At the Daniel den Hoed Cancer Center only patients under the age of 70 were asked to participate. Of the invited patients, 681 (96.5%) were willing to participate. From 1996 to July 2002, 1,152 patients participated and were entered into the database. The Medical Review Board of the Centers approved the study.

Apart from age of diagnosis, these index patients were unselected for age of breast cancer diagnosis, family history for cancer and history of breast cancer screening. The mean age of the index patients was 52.7 (SD 11.1). Index patients younger than 70 years in the study were comparable with those registered in the Comprehensive Cancer Center of The South-West Netherlands during the years 1996–1999, with regard to age distribution (data not shown). For index patients older than 70 years, our sample was comprised of few cases when compared to the almost complete cancer registry of the region. This is mainly due to the exclusion of these patients in the Daniel den Hoed Cancer Center. In first-degree relatives of index patients in our series, the overall breast cancer incidence was twice as high as in the general population, agreeing with the excess risk reported in other population-based studies [2,3]. For 980 index patients, information regarding breast cancer screening history was available. Overall, 632 (64.5%) patients participated in any kind of screening program. 541 (85.6%) participated in the National Breast Cancer Screening Program (biannual mammography), 42 patients (all above the age of 50; 6.6%) participated in both the National Breast Cancer Screening Program and a High Risk Screening Program (annual mammography), and 49 (7.8%) participated in a High Risk Screening Program. In the majority of these screened women, breast cancer was diagnosed based on symptoms and not based on a finding in the screening program, namely in 286 (52.7%), 33 (67.3%) and 31 (73.8%) cases, respectively.

For each index patient the following disease characteristics were recorded: age at diagnosis of first breast cancer, the occurrence of bilateral breast cancer, and any other diagnosis of cancer. Based on interviews with the index-patients, for each index patient a detailed family history of breast cancer regarding the occurrence of malignancies in first- and second-degree relatives was assessed, and a pedigree was drawn. For each relative, the current age or, if deceased, age of death was assessed. For each relative affected with cancer, the age at diagnosis and the type of cancer (e.g. breast, ovary, and prostate cancer) was registered. Medical records of relatives were not checked, so we were not able to assess types of breast cancer in the family, or to discern bilateral breast cancer from breast cancer recurrences in family relatives besides the index patient. Therefore, the distinction between invasive breast cancer and DCIS, or unilateral and bilateral breast cancer was only assessed for the index patients.

### Study population

The original cohort contained 1,152 index patients of whom 286 patients did not have sisters. These patients and their families could not be included in the study. The remaining 866 index patients had 1,987 sisters. These 1,987 women with and without breast cancer formed the study population.

### Analyses

We analyzed the impact of a family history of cancer on the early onset of breast cancer in 1,987 women (sisters). For each of these women it was determined whether or not they had been diagnosed with breast cancer and, if so, at what age. If they were not diagnosed with breast cancer and still alive, their age at the time of the interview with the index patient was assessed. If they were not diagnosed with breast cancer and deceased, their age of death was assessed. Additionally, their family history was analyzed. The following were considered to be first-degree relatives of these women: the affected sister (index patients), mothers, and daughters. To avoid dependency between women in families with more than one sister, sisters with breast cancer other than the index patient were excluded from the family history of the women under study. To explain, suppose a family with three sisters, in which the index has a diagnosis of breast cancer as well as one of the other sisters. All sisters, so both a sister affected with breast cancer and a sister unaffected with breast cancer, were included in the analysis as an outcome, with only the information regarding breast cancer in the index patient (the third sister), mother, daughters and 2nd degree relatives as predictors. The following were considered to be second-degree relatives: grandmothers, aunts (sisters of mother or father), and daughters of the affected sister. Early onset of breast cancer was defined as breast cancer diagnosed before the age of 50 or breast cancer diagnosed before the age of 30.

Family histories included the number of cases of breast cancer in first and second-degree relatives, the age at diagnosis of the first breast cancer, ovarian cancer, the combination of ovarian and breast cancer in one person, prostate cancer under the age of 60, and bilateral breast cancer or DCIS in the index patient.

First, we examined the effect of the various family histories on age at breast cancer diagnosis in the women by using a Kaplan-Meier model. Log rank tests were performed. Age of the women was taken as time variable. Follow-up time was censored at their age of the diagnosis of first breast cancer, their age at the moment of the interview with the index patient or the age when dying.

Second, by using Cox-regression, the age of first breast cancer diagnosis was univariate modeled as a function of the various types of family histories (covariates) yielding hazard ratios. Due to low prevalence, the hazard ratios could be considered as relative risks. To test the assumption of proportional hazards, an interaction term of a covariate and a time-dependent covariate was added [10]. A significant effect of that interaction term denotes the presence of a time-dependent effect and thus a violation of the proportional hazards assumption. As the estimated hazard ratios were not constant over time, we performed Cox-regression with a time-dependent effect, with age at breast cancer diagnosis in the women under study as the dependent variable. For each covariate, a model was built including this covariate and an interaction term of a covariate and a time-dependent covariate. Hazard ratios for the women's chances of developing breast cancer at age 30 and an estimate of the relative change with increasing age (a period of 10 years) of these hazard ratios were calculated.

Then, a multivariate analysis was performed, by using a Cox-regression model again with a time-dependent effect, and with age at breast cancer diagnosis in the women under study as the dependent variable. The model was built in a stepwise fashion, including variables that improved the overall fit of the model with statistical significance (p < 0.05). Again, a hazard ratio for the women's chances of developing breast cancer at age 30 and an estimate of the relative change with increasing age (a period of 10 years) of this hazard ratio was calculated.

The developed model was used to calculate a model score, based on the family history characteristics included in the multivariate model. Each family history characteristic yielded one point in this model. Then we verified how many women with breast cancer were identified correctly, by using different cut-off values of the model score. Furthermore, positive and negative predictive values of the model, with regard to an early onset of breast cancer in the women under study, were calculated.

## Results

### Study population

**Figure 1:**
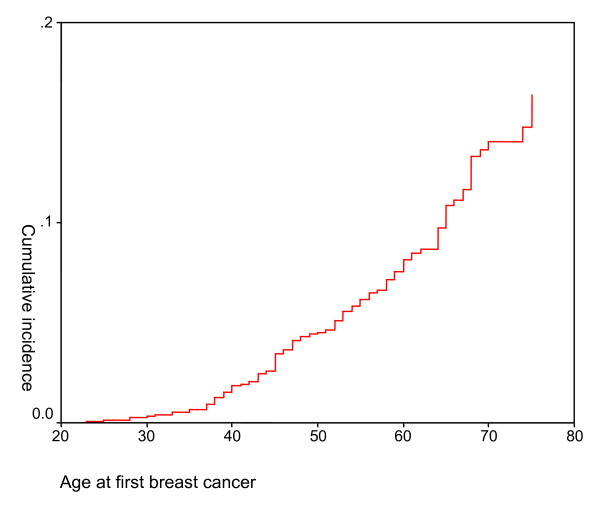
**Age at onset of first breast cancer among the women under study**. x-axis: Age at first breast cancer. y-axis: Cumulative incidence.

Of the 1,987 women under study, 136 (6.8%) had been diagnosed with breast cancer. The incidence of breast cancer increased with age (Figure 1).

### Family history and age of breast cancer onset

The 1,987 women included in the study had 32,791 first or second-degree relatives in total. A higher risk of breast cancer among these women was statistically significantly related to having at least 2 cases of female breast cancer in first or second-degree relatives, at least 1 case of early female breast cancer (i.e. under the age of 50 or 40), or bilateral breast cancer (Table 1). These effects were stronger for women under the age of 50 with breast cancer. The presence of one of these characteristics more than doubled the breast cancer risk at age 30 (Table 2). This risk decreased with increasing age.

**Table 1:** Family histories* of all women under study (N = 1,987), of all women with breast cancer (n = 136) and of all women with breast cancer under the age of 50 (n = 73)

Presence of Family characteristics	All women	Women with breast cancer			
		
		Breast cancer at any age	Log rank; P	Breast cancer under age 50	Log rank; P
	N (%)	N (%)		N (%)	

Total	1987 (100)	136 (6.8)		73 (3.7)	

≥ 2 cases of female breast cancer in first or second-degree relatives	614 (30.9)	54 (39.7)	7.39; P = .007	31 (5.0)	5.02; P = .03
≥ 2 cases of female breast cancer in first-degree relatives	165 (8.3)	20 (14.7)	5.23; P = .02	13 (17.8)	9.85; P = .002

≥ 1 case of female breast cancer under age 50	789 (39.7)	52 (6.6)	12.47; P = .0004	35 (4.4)	7.85; P = .005
≥ 2 cases of female breast cancer under age 50	113 (5.7)	10 (8.8)	1.92; P = .17	10 (8.8)	12.33; P = .0004
≥ 1 case of female breast cancer under age 40	231 (11.6)	18 (7.8)	12.01; P = .0005	12 (5.2)	8.52; P = .004
≥ 2 cases of female breast cancer under age 40	10 (0.5)	-	-	-	-
≥ 1 case of female breast cancer under age 30	33 (1.7)	1 (3.0)	-	1 (3.0)	-

Male breast cancer	12 (0.6)	1 (8.0)	-	1 (8.0)	-

Bilateral breast cancer in affected sister (index patient)	104 (5.2)	16 (15.4)	13.94; P = .0002	11 (10.6%)	15.00; P = .0001

Ovarian cancer	54 (2.7)	5 (9.3)	1.93; P = .16	3 (5.5%)	1.23; P = .27

Breast and ovarian cancer in one person	8 (0.4)	2 (25.0)	12.05; P = .0005	2 (25%)	14.00; P = .0002

Prostate cancer under age 60	43 (2.1)	1 (2.3)		1 (2.3%)	

DCIS in affected sister (index patient)	169 (8.5)	15 (8.9)	0.97; P = .32	7 (4.1%)	0.04; P = .84

* To avoid dependency between women in families with more than one sister, sisters other than the index patient were excluded from the family history of the women under study.

**Table 2:** The effect of the various patterns of breast cancer in the family on the age of onset of breast cancer in the women under study (univariate) *

Family characteristics	Relative risk at age 30**	95% C.I. relative risk at of age 30	Change of relative risk per 10 years ***	Overall Significance ****
≥ 2 cases of female breast cancer in first or second-degree relatives (yes vs. no)	2.20	1.15–4.22	0.851	P = .013
≥ 2 cases of female breast cancer in first-degree relatives (yes vs. no)	4.28	1.89–9.70	0.624	P = .001

≥ 1 case of female breast cancer under age 50 (yes vs. no)	2.82	1.43–5.57	0.800	P = .001
≥ 2 cases of female breast cancer under age 50 (yes vs. no)	5.81	2.16–15.60	0.825	P = .002
≥ 1 case of female breast cancer under age 40 (yes vs. no)	3.21	1.36–7.56	0.825	P = .001

Bilateral breast cancer in affected sister (index patient) (yes vs. no)	4.00	1.55–10.32	0.784	P = .000

Ovarian cancer (yes vs. no)	2.72	0.58–12.83	0.801	P = .294

DCIS in affected sister (index patient) (yes vs. no)	0.89	0.30–2.63	1.184	P = .412

* Due to small numbers, results regarding ≥ 2 cases under age 40 and ≥ 1 or 2 case under age 30 of female breast cancer, male breast cancer, breast and ovarian cancer in one person and prostate cancer under age 60 are not presented.

** Due to low prevalence, the hazard ratio can be considered as a relative risk.

*** The relative risk at age 30 can be multiplied with this factor for calculating the relative risk at age 40, age 50 and so on.

**** Model significance was based on the likelihood ratio of the final model as compared to the O-model without any covariate.

In this population, having at least 2 cases of female breast cancer under the age of 40, having at least 1 case of female breast cancer under the age of 30, or having male breast cancer or prostate cancer under the age of 60 was rare (Table 1). Ovarian cancer and DCIS were not statistically significant associated with an early age at onset of breast cancer in the women under study (Table 1).

Four variables (at least 2 cases of female breast cancer in first-degree relatives (yes/no), at least 2 cases of breast cancer in first or second-degree relatives under the age of 50 (yes/no), at least 1 case of breast cancer under the age of 40 in a first or second-degree relative (yes/no), and any case of bilateral breast cancer (yes/no)) contributed to the presence of breast cancer at 30 years of age, both independently and with statistical significance (Table 3). Of these four family characteristics, the highest hazard ratio for developing breast cancer was related to bilateral breast cancer (HR: 3.47) and the lowest was related to having at least one case of female breast cancer under the age of 40 (HR: 2.06). All the hazard ratios decreased with increasing age, except the one related to having at least one case of female breast cancer under the age of 40 years.

**Table 3:** The effect of the various patterns of breast cancer in the family on the age of onset of breast cancer in the women under study (multivariate)

Family characteristics	Relative risk at age 30 *	95% C.I. of relative risk at age 30	Change of relative risk per 10 years **	Overall Significance ***
≥ 2 cases of female breast cancer in first or second-degree relatives (yes vs. no)	3.09	1.28–7.44	0.745	
≥ 2 cases of female breast cancer under age 50 (yes vs. no)	3.36	1.12–10.08	0.489	
≥ 1 case of female breast cancer under age 40 (yes vs. no)	2.06	0.83–5.12	1.000	
Bilateral breast cancer in affected sister (index patient) (yes vs. no)	3.47	1.33–9.05	0.834	< .001

Model score ****				
0 *****	1			
1	2.01	(0.89–4.55)	0.942	
≥ 2	10.62	(4.22–26.72)	0.430	<.001

* Due to low prevalence, the hazard ratio can be considered as a relative risk.

** The relative risk at age 30 can be multiplied with this factor for calculating the relative risk at age 40, age 50 and so on.

*** Model significance was based on the likelihood ratio of the final model as compared to the O-model without any covariate.

**** Model score based on the family characteristics included in the multivariate model, each characteristic present yields one point.

***** Reference category.

### Model score

Based on family history characteristics included in the multivariate model, a model score was calculated; each family history characteristic yielded one point in this model. As scores of three and four were rare (seven and three cases respectively), the score was recoded into '0', '1', and '2 or more'. Scores of 1 and higher were registered for about one-quarter of all (affected and disease-free) women, for about one-third of the women with breast cancer (regardless of age), for 40% of the women with breast cancer under the age of 50, and for 60% of the women with breast cancer under the age of 30 (Table 4).

**Table 4:** The model scores

Model score	Number of selected women	Women with breast cancer	Women with breast cancer under age 50	Women with breast cancer under age 30
0	1496 (75.3%)	91 (66.9%)	44 (60.3%)	2 (40%)
1	382 (19.2%)	31 (22.8%)	17 (23.3%)	2 (40%)
≥ 2	109 (5.5%)	14 (10.3%)	12 (16.4%)	1 (20%)

### Predictive value of model score

**Figure 2:**
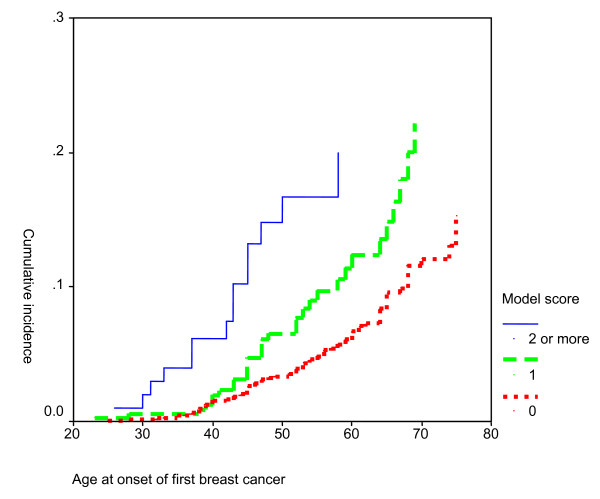
**Age at onset of first breast cancer among the women under study related to the model score**. x-axis: Age at onset of first breast cancer. y-axis: Cumulative incidence

As the model score increased, the age of breast cancer diagnosis decreased (Figure 2). The hazard ratio for developing breast cancer at 30 years of age was notably related to a model score of 2 or more (10.62; Table 3). With increasing age of the women, this hazard decreased sharply (hazard ratio at age 40: 4.56; at age 50: 1.96).

The positive predictive values for model scores of '1 or more' and '2 or more' for breast cancer before the age of 30 were 0,5% and 0.9% respectively, 4% and 11% respectively for breast cancer before the age of 50, and 8% and 13% respectively for breast cancer before the age of 70 (Table 5). The negative predictive value of the model scores varied from nearly 100% to 94%. The concordance index varied from 77% tot 94%.

**Table 5:** The positive and negative predictive value of the model scores and the concordance index

Model score	Positive predictive value	Negative predictive value	Concordance index*
	Outcome: Woman with breast cancer under age 70		

1	8.1%	93.9%	77.4%
≥ 2	12.8%	93.9%	89.4%

	Outcome: Woman with breast cancer under age 50		

1	4.4%	96.5%	78.8%
≥ 2	11.0%	96.8%	92.0%

	Outcome: Woman with breast cancer under age 30		

1	0.5%	99.8%	80.7%
≥ 2	0.9%	99.8%	94.4%

* Percentage of correctly classified cases.

## Discussion and Conclusion

The aim of this study was to examine whether a family history of cancer is useful in predicting early-onset female breast cancer. This was studied in a population-based setting as compared to a high-risk clinic. Family history was indeed a strong indicator of an early diagnosis of breast cancer, and it was most strongly related to breast cancer diagnosed before the age of 50. Factors related to family histories that were connected to a younger age at breast cancer diagnosis were: (1) at least 2 cases of female breast cancer in first-degree relatives (yes/no), (2) at least 2 cases of female breast cancer in first or second-degree relatives under the age of 50 (yes/no), (3) at least 1 case of breast cancer in a woman younger than 40 years in a first or second-degree relative (yes/no), and (4) any case of bilateral breast cancer (yes/no). These four factors were included in a model score, each representing one point in this model. When at least two of these characteristics were present, we found a hazard ratio for developing breast cancer at the age of 30 of 10.62. With increasing age, this hazard decreased sharply (hazard ratio at age 40: 4.56; at age 50: 1.96). However, due to the low prevalence of early breast cancer in the population, the positive predictive value of the presence of at least 2 of the 4 characteristics (a model score of 2 or more) was 13% for breast cancer before the age of 70, 11% for breast cancer before the age of 50, and 1% for breast cancer before the age of 30. The negative predictive value was respectively, 94%, 97% and nearly 100%.

In most guidelines the prerequisite for starting breast cancer surveillance before the age of 50 is at least one first-degree relative diagnosed with breast cancer under the age of 40 [4,5] or 50 [6,7]. Additional criteria are: more than one relative with breast cancer, relatives with bilateral breast cancer, ovarian cancer, male breast cancer, or prostate cancer before the age of 60 [8]. In our study, at least 2 cases of female breast cancer in first-degree relatives, or having at least 1 case of breast cancer in a woman younger than 40 years in a first or second-degree relative were associated with early onset of breast cancer. This is in line with Claus tables who models age of onset of breast cancer as a function of family history of breast among first degree relatives [11]. As compared to the Claus tables, we added the criterion having at least 2 cases of female breast cancer in first or second-degree relatives under the age of 50. In our study, having one first-degree relative with breast cancer before the age of 50 was not associated with early onset breast cancer. This is also in line with the Claus tables [11].

In our study, male breast cancer, DCIS, ovarian cancer, and prostate cancer under age 60 in the family were not associated with early onset of breast cancer. In addition, we found a lower prevalence of ovarian cancer, early onset prostate cancer and male breast cancer than in other studies on breast cancer risk assessment [12-14]. A first explanation might be that these types of cancer are not very well known among family members and that these cancers may be underreported in the original interviews with the index patient, with regard to the family history of cancer. All data regarding family history were based on interviews with the index patients; medical records of relatives with malignancy were not checked. This can be considered a valid method insofar as family history is limited to breast cancer among first-degree relatives [15]. Data regarding ovarian and prostate cancer and data regarding second-degree relatives might be underreported [16]. As a consequence, our study had not enough power to analyze whether presence of male breast cancer or ovarian cancer is useful in predicting early-onset female breast cancer.

A second explanation might be, that these types of cancer are more frequent in the study populations of mentioned studies, than could be expected in the general population, as these studies focused on the prediction of breast cancer risk using estimates on the prevalence and penetrance of BRCA1 and BRCA2 mutations. As ovarian cancer, early onset prostate cancer and male breast cancer are associated with such mutations [17,18], the study populations of these studies may have included high risk groups more frequently than low risk groups.

Bilateral breast cancer is generally considered an indicator of a genetic susceptibility of breast cancer. However, recent studies showed only a weak association of BRCA1/2 mutation status with bilateral breast cancer [19-21]. In our study bilateral breast cancer is strongly related to early onset of breast cancer. This may indicate that genes other than BRCA1 and BRCA2 are involved in familial clustering of bilateral breast cancer.

We based our analyses on the presence or absence of breast cancer in women who were sisters of breast cancer patients. These breast cancer patients were included as index patients in a cohort study. Apart from age of diagnosis, this series of index patients can be considered representative of women with a diagnosis for breast cancer, unselected for family history of breast cancer. In one of the participating centers, only patients under the age of 70 were asked to participate. This selection on age might give a slight overestimation of the risks found in this study, especially for the older age groups. Due to relatively small number of index cases in the age group of 70 years and over, we were not able to quantify these effects. However, as the prediction of female early onset breast cancer based on a family history of breast cancer will be especially important for younger women, we suppose that the effects are marginal.

91 (9%) affected patients participated in a High Risk Screening Program with annual mammography. Among these women, only one-third of the breast cancers (n = 27) was diagnosed based on a finding in the screening program. We expect that part of the sisters of these affected patients might have participated in a High Risk Screening Program before their sister (the index patient of our study) was diagnosed with breast cancer. Because it is a very small group we do not expect this has influenced the outcomes of the here presented analysis.

In theory, there might be cohort effects regarding the age of onset of breast cancer. We checked the data, but could not find any. As a consequence, there was no indication to control for a cohort effect. Another consequence was that we could consider the outcome (a sister's cancer) as an equivalent to the predictor (the index cancer) and mutual exchangeable, which allowed us to perform the analysis we did.

Including population-based control subjects who did not have a sister with breast cancer would have given us the opportunity to estimate a baseline risk for women without a family history of breast cancer. However, early-onset breast cancer incidence in the absence of any family history for cancer is likely to be quite low, and because it was not related to the main question of this analysis, we decided not to perform such a study.

Ideally, the current study should be carried out in families who test negative for BRCA1/2 and other familial breast cancer genes. On the other hand, the two breast cancer susceptibility genes thus far known, BRCA1 and BRCA2, only explain 15–20% of the familial clustering of breast cancer, and less than 5% of breast cancer overall [9]. As a consequence, for most women with a family history of cancer, their breast cancer risk estimate will be based on their family history and not on their genetic status [22].

Correction for possible bias, due to dependency between women in families with more than one sister, has not been part of this analysis, as sisters other than the index patient were excluded from the family history of the women under study. Supposing there is some dependency, the accuracy (width of the confidence intervals) of the data might be slightly overestimated. However, because the significance levels are far from the 5% level, we do not expect that this has had an influence on our results.

Validating this model is a next step of research and should preferably be performed in another database [23]. Another way of validating this model is to compare it with existing models. However, as mentioned in the introduction, most existing models have been derived from estimates based on data from cancer prone families. The aim of the current study was to work on a model based on a population not selected for family history of (breast) cancer. The most known model based on such a population is the Claus model, which predictions are in line with our results. Though not demonstrated in this study, it has been shown that male breast cancer and ovarian cancer are important covariates in predicting early onset breast cancer [24,25]. Further research is needed to assess the predictive value of DCIS and prostate cancer under the age of 60.

In conclusion: applying family history related criteria could result in the screening of many women who will not develop breast cancer at an early age. Chances of developing early breast cancer are very small, when there is limited family history for (breast) cancer and none or only one of the following criteria is applicable: (1) at least 2 cases of female breast cancer in first-degree relatives; (2) at least 2 cases of female breast cancer in first or second-degree relatives under the age of 50; (3) at least 1 case of female breast cancer under the age of 40 in a first- or second-degree relative; and (4) any case of bilateral breast cancer. If the model score of women would be below 2 points, their risk for developing breast cancer at an early age would be low, and screening at an early age might not be indicated. At age 40 or 50 (depending on the country they live in), these women will be invited to participate in the national screening program. If the model would be used in clinical decision settings, it would be an easy to use method to reassure a large number of women regarding their personal breast cancer risk at an early age and their need to be referred to early screening programs or genetic centers.

## Competing interests

The authors declare that they have no competing interests.

## Authors' contributions

JGMK, PD, RAEMT, and CTMB developed the design of the cohort. CS, EMM K-W, JB, and CJvA made substantial contributions to acquisition of data. GHdB, CEJ, and JCvH did the data analysis and interpretation of data. GHdB drafted the manuscript and all other authors were involved revising the manuscript critically. All authors have given final approval of the version to be published.

## Pre-publication history

The pre-publication history for this paper can be accessed here:


